# Quadrant Asymmetry in Optical Coherence Tomography Angiography Metrics in Ischemic Versus Non-Ischemic Central Retinal Vein Occlusion Eyes

**DOI:** 10.1167/tvst.12.3.30

**Published:** 2023-03-29

**Authors:** Jesse J. Jung, Xavier Chan, Shen Yi Lim, Scott S. Lee, Soraya Rofagha, Quan V. Hoang

**Affiliations:** 1 East Bay Retina Consultants Inc., Oakland, CA, USA; 2 Department of Ophthalmology, University of California, San Francisco, San Francisco, CA, USA; 3 Singapore Eye Research Institute, Singapore National Eye Centre, Duke-NUS Medical School, Singapore; 4 Department of Ophthalmology, Yong Loo Lin School of Medicine, National University of Singapore, Singapore; 5 Department of Ophthalmology, Edward S. Harkness Eye Institute, Columbia University College of Physicians and Surgeons, New York, NY, USA

**Keywords:** central retinal vein occlusion, intraeye, ischemia, optical coherence tomography angiography, quadrant asymmetry

## Abstract

**Purpose:**

To determine whether quadrant asymmetry (QA) of optical coherence tomography angiography (OCTA) metrics differs between non-ischemic versus ischemic central retinal vein occlusion (CRVO).

**Methods:**

Fifty-eight eyes (21 non-ischemic, 10 ischemic CRVO, and 27 contralateral control eyes) underwent 3 × 3 mm spectral-domain OCTA scans with quantification of the superficial retinal layer vessel length density (VLD) and perfusion density (PD). QA, defined as the maximum-minus-minimum value among four parafoveal Early Treatment Diabetic Retinopathy Study (ETDRS) quadrants, was compared by linear regression including fixed effects for each eye.

**Results:**

Mean age was 73.6 ± 11.4 (range 39-88), 73.8 ± 12.4 (range 39–91) and 77.2 ± 9.83, (range 60–88); and QA was 3.46 ± 1.76, 3.14 ± 1.57, and 4.88 ± 2.42 for VLD and 0.072 ± 0.038, 0.062 ± 0.036, and 0.11 ± 0.056 for PD for control, non-ischemic, and ischemic, respectively. QA was significantly higher in ischemic (0.109 ± 0.056) than non-ischemic CRVO eyes (0.062 ± 0.036; *P* = 0.02) and control eyes for PD (0.072 ± 0.038; *P* = 0.03). QA was also greater in ischemic (4.875 ± 2.418) than non-ischemic CRVO (3.141 ± 1.572) for VLD (*P* = 0.04). In terms of identifying which particular quadrant is most affected by ischemia, multivariate regression analysis comparing intra-quadrant effect on the presence of ischemia versus non-ischemia showed no quadrant was significantly affected (*P* > 0.05 for all quadrants).

**Conclusions:**

Ischemic CRVO increases intraeye QA of OCTA metrics when compared to non-ischemic CRVO and control eyes. No specific ETDRS quadrant appears to be more affected.

**Translational Relevance:**

This work uses an intraeye method to delineate between ischemic and non-ischemic CRVO by OCTA imaging, overcoming inter-eye variables encountered in clinical care.

## Introduction

Central retinal vein occlusion (CRVO) is the second most common retinal disease, with a worldwide prevalence of 0.13% or 4.67 million of the population being affected.^1^ Significant hypoxia or ischemia may occur resulting in complications leading to vision loss such as macular edema and neovascularization.^2^^–^^4^ CRVO may be categorized into non-ischemic (perfused) and ischemic (non-perfused) states based on the perfusion area in the capillaries and best-correct visual acuity, each with its own clinical features, prognosis, and therapies.^5^^,^^6^ Non-ischemic CRVO at later stages may also progress to ischemic CRVO.^7^

To distinguish between non-ischemic and ischemic states, various groups have proposed and applied differing approaches, ranging from ophthalmodynamometric differences,^8^ electroretinography,^9^ and biochemical markers such as plasma homocysteine.^10^ Additionally, multimodal imaging with fundus photography, fluorescein angiography (FA), optical coherence tomography (OCT), and OCT angiography (OCTA) have been used to diagnose and differentiate between types of CRVO to further prognosticate and guide treatment. For example, traditionally, FA is used to distinguish between both non-ischemic and ischemic groups based on the amount of peripheral perfusion.^5^ Moreover, FA has also been used to prognosticate with accuracy, visual outcomes in CRVO patients.^11^ Although usage of FA as an imaging technique to assess CRVO patients may seem optimal, limitations such as invasiveness, cost and time, risk of rare adverse allergic reactions caused by injection of an intravenous dye,^12^ and restricted depth resolution to decipher between retinal capillary plexuses and the choroid may render other imaging tools such as non-invasive and dye-free OCT^13^ and its functional extension, OCTA, to be more promising because of optimum imaging of different layers of the retinal vasculature.^14^

OCTA can analyze both the superficial and deep capillary plexuses in CRVO eyes and ascertain the density of vasculature, which is imperative as low vascular densities may be associated with more severe neovascular complications.^15^ With OCTA however, drawbacks include poor fixation leading to motion artifacts, projection artifacts, errors in segmentation, reduced field of view, limited information on permeability or leakage of the vasculature, and inaccurate interpretations of images attributed to intricacies of distinguishing between pseudoflow and true flow.^16^^,^^17^ Moreover, variabilities between eyes and between subjects such as age, astigmatism, axial length, and refractive error may elicit more obstacles when comparing retinal images across different cohorts and may necessitate mathematical calculations to account for variation.^18^^–^^21^

Quadrant asymmetry (QA), a method to compare intraeye variabilities, may be used to address these limitations and examine the differences between Early Treatment Diabetic Retinopathy Study (ETDRS) quadrants in OCTA quantitative metrics. QA has been used by our group in patients with central serous chorioretinopathy and pachychoroid diseased eyes to demonstrate asymmetric outflow in vortex veins, using wide field indocyanine green angiography,^22^ can differentiate the effect of astigmatism on quantitative OCTA metrics,^21^ and distinguish between the level of diabetic retinopathy severity.^23^ Herein, we seek to determine whether intraeye QA of OCTA metrics among the four ETDRS quadrants is affected differently when comparing the insult of a non-ischemic versus ischemic CRVO and healthy control eyes.

## Methods

This observational, retrospective cross-sectional cohort study received institutional review board (IRB) approval from Salus IRB (Austin, TX, USA). This study complied with the Health Insurance Portability and Accountability Act of 1996 and followed the tenets of the Declaration of Helsinki. All individuals signed a written informed consent as part of an imaging study incorporating the use of the Cirrus AngioPlex software (version 10.0; clearance by the US FDA pending; Carl Zeiss Meditec, Inc., Dublin, CA, USA) before participating in the study.

### Participants

During their routine clinical care, individuals were retrospectively identified with a diagnosis of non-ischemic or ischemic CRVO eye (defined as best-corrected visual acuity <20/200 or greater than 10-disc areas of peripheral retinal capillary non-perfusion on angiography)^5^^,^^6^ and both eyes, including contralateral control eyes, were selected for the study.

### OCT Imaging

All images obtained from the eye were taken at a single timepoint before analysis. Briefly, all OCTA images were captured using a spectral-domain OCTA (Cirrus 5000 with AngioPlex; Carl Zeiss Meditec, Inc., Dublin, CA, USA) via an angiography 3 × 3 mm scan pattern with a 245 × 245 resolution and a mean distance of 12.2 µm between each scan and images at 68,000 A-scans per second with a light source with a central wave-length of 840 nm and a full width at half maximum bandwidth of 90 nm. The A-scan depth was 2 mm with an axial resolution of 5 µm and a transverse resolution of 15 µm.^24^ Using the optical microangiography algorithm, en face OCTA images were obtained. Standard OCTA tracking software, accompanied by image centering on the fovea, was utilized to reduce motion artifacts. OCTA scans with signal strength greater than 7 (normal scale 1–10), auto-segmented for the superficial retinal layer (SRL), and uniform illumination exclusive of areas of darkness and without motion artifacts (demonstrated via vessel segment misalignment) were obtained. Additionally, correct identification of the SRL was ensured by stringent examination of each segmentation, to ensure the default segmentation boundaries of the device were accurate. Projection artifacts were removed utilizing the Cirrus AngioPlex software (version 10.0; clearance by the US FDA pending; Carl Zeiss Meditec, Inc., Dublin, CA, USA) before images were analyzed.

Because of concerns of potential segmentation errors, which could impact measurements of the SRL, CRVO eyes with cystoid macular edema involving the central 3 × 3 mm area (of the scan pattern) were excluded from the study. OCT/OCTA images were acquired at presentation and each follow-up, but those included were based on the single time point when there was no macular edema that could confound interpretation of the OCTA quantitative analysis. CRVO eyes with prior or current/ongoing treatment with anti-vascular endothelial growth factor (anti-VEGF) injections such as aflibercept (Regeneron, Tarrytown, NJ, USA), bevacizumab (Genentech, South San Francisco, CA, USA) and/or ranibizumab (Genentech) were included. All eyes included in this study were well-controlled on maintenance or as-needed treatment without exhibiting signs of active cystoid macular edema or vitreous hemorrhage at the time of imaging.

### Quadrant Asymmetry Analysis

**Figure 1.**
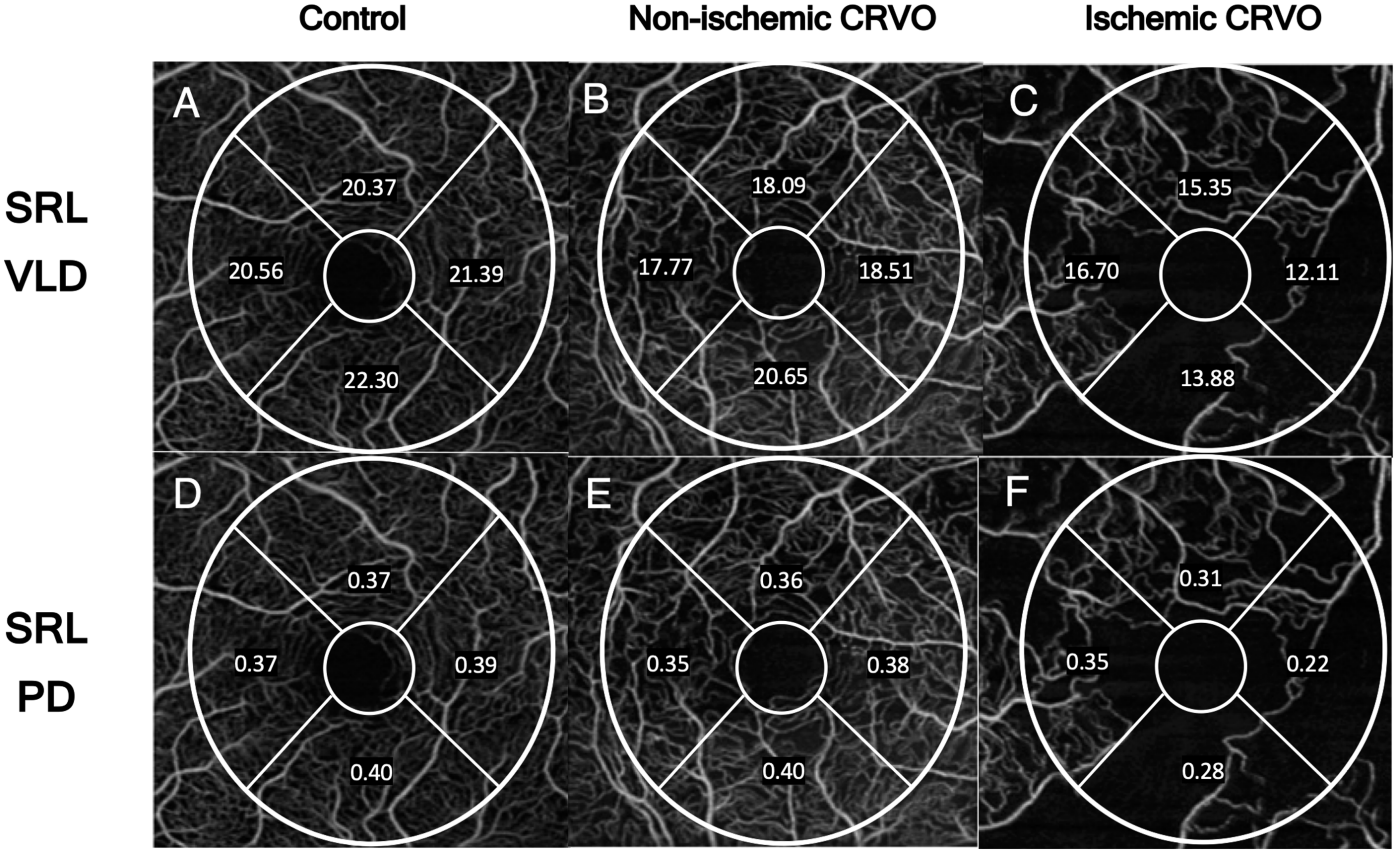
QA of VLD for control, non-ischemic and ischemic central retinal vein occlusion eyes. Quantitative measurements of VLD and PD in the parafoveal ETDRS subfields in spectral domain OCTA images of the SRL. QA values for control (left-most column; A, D), non-ischemic CRVO (middle column; B, E) and ischemic CRVO (right-most column; C, F).

The Cirrus AngioPlex software (version 10.0; clearance by the US FDA pending; Carl Zeiss Meditec, Inc., Dublin, CA, USA) was used to export and analyze OCTA en face images. The ETDRS parafoveal inner ring is defined as a concentric ring with an inner diameter of 1000 µm and an outer diameter of 3000 µm centered at the fovea. This inner ring can be divided into superior, inferior, nasal, and temporal subfields, which constitute the four quadrants assessed in our analysis. The parafoveal vessel length density (VLD) was defined as the total length of perfused vasculature per unit area (mm^−^^1^) and perfusion density (PD), defined as the total area covered by perfused vasculature per unit area (%). The VLD and PD were computed in each of these ETDRS subfields/quadrants for the SRL (Fig. 1).

Quadrant Asymmetry (QA) was calculated as the maximum-minus-minimum (max-min) value of the greatest and the least OCTA SRL metric from each quadrant of a given eye and was used to assess the asymmetry and non-uniformity among four ETDRS quadrants.^23^ Mann-Whitney U test was used to quantify the mean max-min difference in values between ischemic eyes and control combined with non-ischemic eyes via plotting of the cumulative distribution function. By using a multivariate linear regression, and accounting for patient-eye-specific variability with fixed effects, specific quadrants with the greatest quantitative VLD or PD metric in the included eyes could be determined. This analysis was performed on values within a quadrant with dummy variables in order to analyze individual quadrants. The superior quadrant was set as the reference group. All data were analyzed using the Stata 13.0 statistical package (StataCorp LP, College Station, TX, USA). All quantitative values were expressed as the mean with standard deviation or mean with standard error. Data was considered significant when *P* < 0.05.

## Results

Fifty-eight eyes from 31 patients, consisting of contralateral control, non-ischemic, and ischemic were included. Twenty-seven eyes (27 patients) were normal, 21 eyes (20 patients) were non-ischemic, and 10 eyes (10 patients) were ischemic. For the contralateral control eyes, one patient was monocular and 1 patient had bilateral CRVO. At the time of image acquisition for the cross-sectional study, 25 eyes had been previously treated with anti-VEGF. Disease duration and follow-up time did not significantly differ between the ischemic and non-ischemic groups (Table 1).

**Table 1.** Demographics

Characteristic	Control (*n* = 27 Eyes of 27 Patients)	Non-Ischemic (*n* = 21 Eyes of 20 Patients)	Ischemic (*n* = 10 Eyes of 10 Patients)
Age (y), mean ± SD (range)	73.6 ± 11.4 (39–88)	73.8 ± 12.4 (39–91)	77.2 ± 9.83 (60–88)
Last BCVA ETDRS, mean ± SD (range)	26.852 ± 8.452 (20–50)	35.714 ± 15.675 (20–80)	2560.600 ± 6172.150 (126–20,000)
Last logMAR VA, mean ± SD (range)	0.113 ± 0.116 (0–0.4)	0.220 ± 0.167 (0–0.6)	1.419 ± 0.685 (0.9–3)
Number of injections, mean ± SD (range)	—	10.6 ± 8.9(1–34)	14.9 ± 12.2 (2–39)
Average number of weeks per injection, mean ± SD (range)	—	10.3 ± 6.1 (4–23)	7.1 ± 2.3 (5–12)
Mean follow-up (wk) , mean ± SD (range)		113 ± 80.3 (10–341)	155 ± 106.9 (35–311)
Current injection interval (wk), mean ± SD (range)	—	6.6 ± 2.8 (4–12)	9.0 ± 2.7 (6–14)
Central foveal thickness (µm), mean ± SD (range)	264.741 ± 23.335 (193–330)	283.714 ± 31.219 (217–332)	207.700 ± 34.419 (160–250)
Laterality (right eye)	12 (44.4%)	13 (61.9%)	3 (30.0%)
Gender (male)	18 (66.7%)	11 (52.4%)	9 (90.0%)
Lens Status (Phakic)	17 (63.0%)	9 (42.9%)	4 (40.0%)
Treatment (anti-VEFG)	—	16 (76.2%)	9 (90.0%)
CRVO	—	21 (100.0%)	10 (100.0%)

SD, standard deviation; logMAR, logarithm of the minimum angle of resolution; BCVA, best-corrected visual acuity; wk, week.

Baseline demographics comparing 58 eyes of 31 patients of control, non-ischemic and ischemic. The BCVA value is the denominator and is presented as the mean with 20/26.852 for control, 20/35.714 for the non-ischemic, and 20/2560.600 for ischemic eyes.

Baseline characteristics of included subjects are summarized in Table 1. Comparing baseline demographic variables between the control, non-ischemic and ischemic eyes, average age was 73.6 ± 11.4 (range 39–88) in the control group, 73.8 ± 12.4 (range 39–91) in the non-ischemic group and 77.2 ± 9.83 (range 60–88) in the ischemic group. QA values for the control, non-ischemic and ischemic are presented as mean ± standard deviation (Table 2). Comparing QA by Mann-Whitney U test (mean ± standard error), ischemic (0.109 ± 0.056) was significantly higher than non-ischemic CRVO eyes (0.062 ± 0.036; *P* = 0.02) and control eyes for PD (0.072 ± 0.038; *P* = 0.03). QA was also greater in ischemic (4.875 ± 2.418) than non-ischemic CRVO (3.141 ± 1.572) for VLD (*P* = 0.04) by Mann-Whitney U test. No significant difference was observed for VLD between ischemic and control, and VLD and PD between control and non-ischemic (*P* > 0.05 for all comparisons, Table 3).

**Table 2.** QA Values for Control, Non-Ischemic, and Ischemic

Image Type	Control (*n* = 27)	Non-Ischemic (*n* = 21)	Ischemic (*n* = 10)
SRL VLD	3.459 ± 1.762	3.141 ± 1.572	4.875 ± 2.418
SRL PD	0.072 ± 0.038	0.062 ± 0.036	0.109 ± 0.056

SD, standard deviation.

Data are presented as mean ± SD. PD is defined as the total area covered by perfused vasculature per unit area (%); VLD is defined as the total length of perfused vasculature per unit area (mm/mm^2^).

**Table 3.** Images Comparing Quadrant Asymmetry Between Control, Non-Ischemic, and Ischemic

Image Type	Condition (No. of Images)		Mean Difference in QA (Mean ± SE)	*P* Value
SRL VLD	Ischemic (*n* = 10)	Non-ischemic (*n* = 21)	1.734 ± 0.721	0.039^*^
SRL PD	Ischemic (*n* = 10)	Non-ischemic (*n* = 21)	0.047 ± 0.016	0.022^*^
SRL VLD	Ischemic (*n* = 10)	Control (*n* = 27)	1.416 ± 0.722	0.084
SRL PD	Ischemic (*n* = 10)	Control (*n* = 27)	0.037 ± 0.016	0.034^*^
SRL VLD	Control (*n* = 27)	Non-ischemic (*n* = 21)	0.318 ± 0.489	0.636
SRL PD	Control (*n* = 27)	Non-ischemic (*n* = 21)	0.010 ± 0.011	0.322

SE, standard error.

Images were compared for QA between (1) ischemic against non-ischemic, (2) ischemic against control, and (3) control against non-ischemic for SRL VLD and PD. Mean difference was calculated by subtracting the second group from the first group. Data are presented as mean ± SE. Bold values (*) denote significance set at *P* < 0.05. PD is defined as the total area covered by perfused vasculature per unit area (%); VLD is defined as the total length of perfused vasculature per unit area (mm/mm^2^).

In terms of identifying which particular quadrant is most affected by ischemia, multivariate regression analysis was utilized to compare the intra-quadrant effect on the presence of ischemia vs. non-ischemia. Using the superior quadrant as the reference group, no significant difference was found between quadrants for VLD and PD (*P* > 0.05 for all quadrants, Table 4).

**Table 4.** Multivariate Regression Analysis Comparing Intra-Quadrant Effect on Non-Ischemic and Ischemic

Quadrant	Coefficient	Standard Error	*P* Value
SRL VLD			
Superior	Reference group	**—**	**—**
Inferior	2.069	1.149	0.073
Nasal	0.783	0.879	0.374
Temporal	0.426	1.013	0.675
SRL PD			
Superior	Reference group	**—**	**—**
Inferior	0.045	0.025	0.075
Nasal	0.014	0.019	0.479
Temporal	0.002	0.022	0.933

Multivariate linear regression analysis was performed comparing SRL VLD and PD between quadrants (superior, inferior, nasal, temporal) between non-ischemic and ischemic, with superior quadrant set as the reference group and controlling for patient eye specific fixed effects. Bold values (*) denote significance set at *P* < 0.05. PD is defined as the total area covered by perfused vasculature per unit area (%); VLD is defined as the total length of perfused vasculature per unit area (mm/mm^2^).

**Figure 2.**
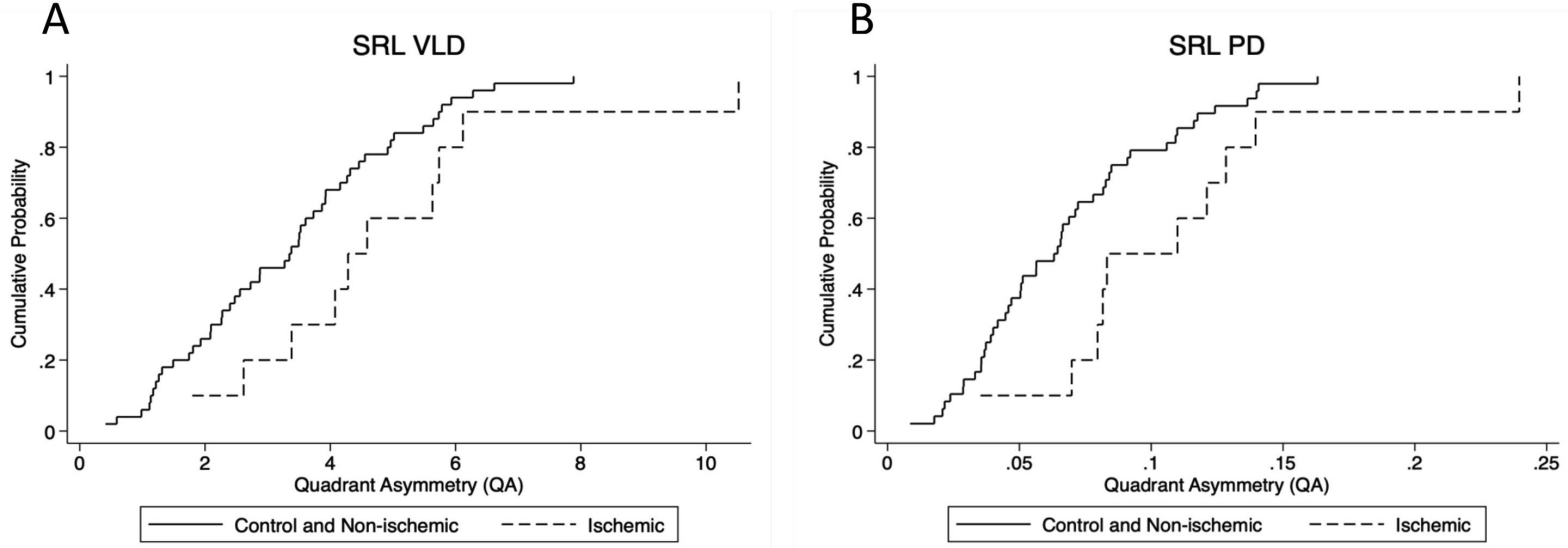
CDF graph of QA (Maximum-minus-Minimum) for control and non-ischemic group compared to ischemic group. QA was calculated by subtracting the quadrant with the minimum value from the quadrant with the maximum value for each individual eye. CDF plot showed that ischemic group had greater QA than control and non-ischemic group across SRL VLD and PD. PD is defined as the total area covered by perfused vasculature per unit area (%); VLD is defined as the total length of perfused vasculature per unit area (mm/mm^2^).

**Figure 3.**
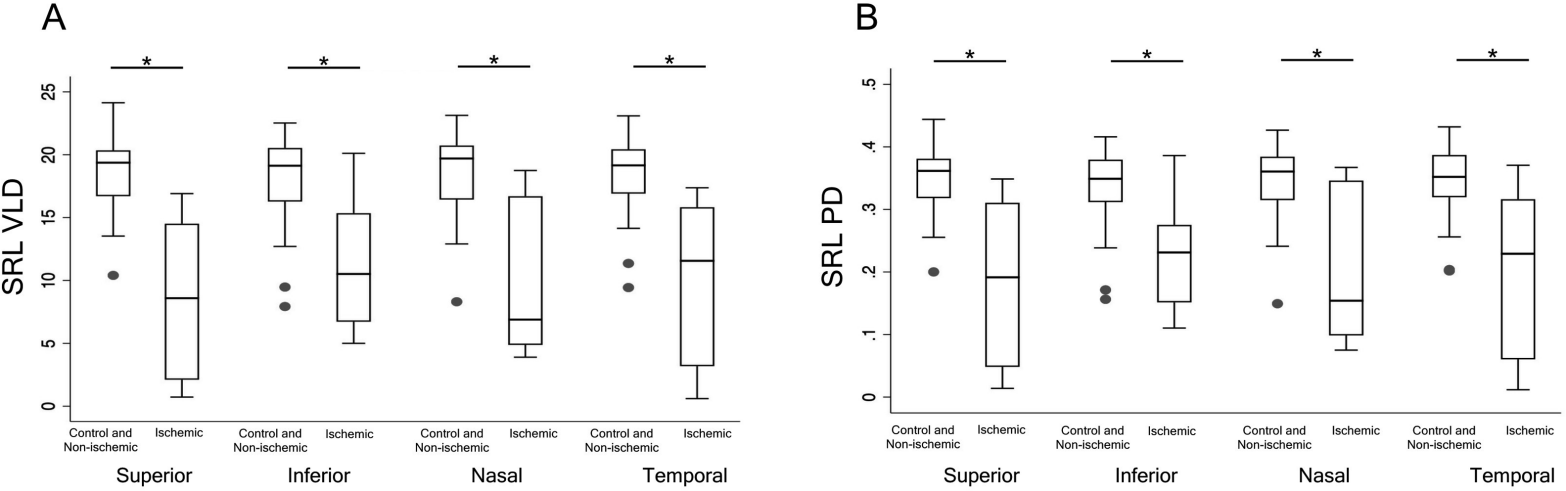
Intra-quadrant comparison of images for the superficial retinal layer vessel length density and perfusion density for control and non-ischemic group compared to ischemic group. *Asterisk* denotes significance set at *P* < 0.05. PD is defined as the total area covered by perfused vasculature per unit area (%); VLD is defined as the total length of perfused vasculature per unit area (mm/mm^2^).

The cumulative distribution function (CDF) plot in Figure 2 shows the difference in distribution of QA values in control and non-ischemic against ischemic, for both VLD and PD. Distribution for ischemic (dotted line) are right-shifted relative to control and non-ischemic eyes (solid line) for VLD and PD, which indicates that the ischemic group has QA values that are higher relative to control throughout the distribution. Distribution for the control group also showed a lower variability in QA values with a steeper CDF plot for VLD and PD. For VLD and PD within the ischemic group, outlier value exists as seen by the heavy tail end of its CDF from QA values 6 to 10 for VLD and 0.15 to 0.25 for PD. Intra-quadrant comparison in Figure 3 of VLD and PD showed significant difference in values between control and the ischemic group for all quadrants of VLD and PD (*P* < 0.05 for all comparisons, Mann-Whitney U test).

## Discussion

In this study, we assessed the accuracy of an intraeye comparison using QA among the ETDRS inner ring subfield OCTA metrics to predict the severity of ischemia associated with CRVO. When comparing ischemic CRVO to non-ischemic eyes, QA was significantly higher for both VLD and PD. Additionally, QA in ischemic CRVO was significantly greater than control eyes (fellow healthy eyes) for PD OCTA metric. However, when comparing control eyes to eyes with non-ischemic CRVO, QA for the VLD and PD was not significantly different (Table 3). Multivariate regression analysis demonstrated that in general, there was not a significant quadrant for both OCTA metrics that was more asymmetrically affected with the presence of non-ischemic and ischemic CRVOs (Table 4).

OCTA has enhanced our ability to quantify the level of ischemia in CRVO. Previous studies have demonstrated worsening capillary dropout in both the SRL and DRL in CRVO.^25^^–^^27^ However, variables between eyes, as mentioned previously, may lead to inaccurate comparisons of OCTA metrics across eyes of a population. For example, to correct for the variability in magnification with differing axial lengths, the Littman and modified Bennett formulas are required.^19^ As a result of these intereye differences, difficulties in replication of longitudinal quantitative OCTA data may arise when comparisons are done between diverse populations of eyes such as eyes with high myopia.^28^ To possibly mitigate for these issues, QA uses a comparison of intraeye metrics including the difference between the ETDRS parafoveal inner ring quadrants (with statistical adjustment for fixed effects for each dataset with linear regression analysis), as shown by our previous studies.^21^^–^^23^ Reviewing the literature, no previous studies have demonstrated the effect of an intraeye OCTA measure in CRVO eyes. Herein, we similarly used QA to demonstrate that we can accurately distinguish between non-ischemic and ischemic CRVO based on an increasing QA (Tables 2 and 3).

It is important to delineate between non-ischemic and ischemic CRVO, the latter being the more severe form with high risks for neovascular complications such as vitreous hemorrhage and neovascular glaucoma. These complications may lead to significant loss of vision if left untreated, thus warranting more frequent follow-ups in clinic.^29^ As such, distinguishing between the two different types of CRVO would allow physicians to better decide on and measure efficacies of anti-VEGF treatment strategies,^30^^,^^31^ the functional benefit of different anti-VEGF agents such as aflibercept, ranibizumab, and bevacizumab,^32^ the possible need for treatment with panretinal photocoagulation for severely affected eyes, and potential long-term prognosis and risk for developing neovascular complications. Analyzing the predictive value of QA, the distribution of QA values showed that control and non-ischemic was lower than ischemic (Fig. 2) across the board. When intra-quadrant comparison was done (Fig. 3), there was significant difference between control and non-ischemic group when compared with ischemic group. There was no difference in QA between non-ischemic and control eyes for both OCTA metrics. Control and non-ischemic CRVO did not differ greatly as eyes with non-ischemic CRVO eyes may not develop enough ischemic burden that would lead to complications such as neovascularization or significant macular ischemia affecting long-term vision. However, we observed significantly greater QA in ischemic eyes compared to both control and non-ischemic eyes, which may be expected given that non-ischemic CRVO may not have as much of an ischemic impact on the capillary plexuses, and the potential threshold of ischemic disease burden may be predictable based on the QA between parafoveal quadrants on a 3 × 3 spectral-domain OCTA. Quadrant asymmetry potentially provides a more accurate measure to follow among large cohorts and longitudinally, thereby avoiding the variabilities that may be inherent in intereye quantitative comparisons.

Multivariate regression analysis comparing intra-quadrant effect on the presence of non-ischemic and ischemic CRVO eyes, with superior quadrant as the reference group, showed that there is no significant difference between quadrants. Based on this regression analysis, in eyes with more ischemic damage from CRVO, the capillary dropout may randomly occur throughout the parafoveal region. Previous studies have shown that flow in the deep capillary plexus is initially affected, with reduced branch numbers, vessel density, and vessel tortuosity.^33^^–^^35^ With more severe ischemic insult, this leads to further loss of both superficial and deep plexuses and this may occur randomly throughout the parafoveal macula.

Limitations of this study include the relatively small sample size found in each category, especially the ischemic CRVO group. Additionally, the research algorithm available only quantitatively analyzed the SRL, and these factors may have limited the statistical significance of the QA analysis with VLD when comparing the ischemic versus control eyes. Previous studies have demonstrated that the deep capillary plexuses appears to be more affected in CRVO compared to the superficial capillary plexuses and further analysis with QA for the deep retinal layers may further elucidate if this metric can further predict the differences in CRVO eyes.^33^^,^^35^ Furthermore, several eyes in both the non-ischemic and ischemic CRVO groups, were previously treated with anti-VEGF. However, imaging was only performed when the eyes showed no active cystoid macular edema. As previously demonstrated by various studies, anti-VEGF treatment has minimal effect on the overall capillary density, neither increasing nor decreasing the quantitative metrics.^36^^,^^37^ Acquisition of OCTA images is also affected by artifacts, such as segmentation errors, projection artifacts, and poor focus, which could modify measurements of both VLD and PD. As such, we accounted for these limitations by only selecting centered images of high quality and signal strength and excluded images which had poor acquisition parameters. Last, as this is a retrospective cross-sectional study utilizing a single spectral-domain OCTA system (Angioplex, Carl Zeiss Meditec Inc., Dublin, CA), OCTA images captured in this study was performed at a single point in time. Therefore, this may affect our ability to extrapolate our results gathered in this study to other commercial devices, but based on the results of this study though, intraeye QA should be a technique that can accurately delineate between ischemic and non-ischemic CRVO regardless of the time point of image acquisition or instrument used. Furthermore, although the risk of macular ischemia is more likely in eyes with longer duration of disease and with a higher burden of anti-VEGF treatment,^38^ because QA is an intraeye metric comparing quadrants within the same eye, it is more robust to effects over time, in contrast to studies that compare uncorrected values of OCTA metrics longitudinally. There is, of course, the possibility that given the likely correlation between duration of disease and presence of ischemia that the QA changes we find at this single point in time in ischemic versus non-ischemic CRVO is partly attributable to disease duration. Moreover, future studies should incorporate comparisons and analysis between different devices to prove this hypothesis and elucidate the potential benefit of using this intraeye OCTA quantitative metric to identify the progression of the disease from non-ischemic to ischemic.

Despite limitations listed above, our study accurately utilized QA as an intraeye quantitative OCTA metric to distinguish between control, non-ischemic, and ischemic CRVO. QA can potentially overcome the confounding effects of intereye variables such as age, axial length, and refractive error. Moreover, QA is easy to acquire, by simple statistical analysis of quantitative metrics routinely obtained within ETDRS quadrants in OCTA images. By improving intraeye en face OCTA image analysis and reproducibility of OCTA-based CRVO screening, increased reliability of disease monitoring may be of benefit to both clinicians and patients in the future.
